# Preliminary Study for AUV: Longitudinal Stabilization Method Based on Takagi-Sugeno Fuzzy Inference System †

**DOI:** 10.3390/s21051866

**Published:** 2021-03-07

**Authors:** Enrico Petritoli, Cipriano Bartoletti, Fabio Leccese

**Affiliations:** Science Department, Università degli Studi “Roma Tre”, Via della Vasca Navale n. 84, 00146 Rome, Italy; enrico.petritoli@uniroma3.it (E.P.); cipriano.bartoletti@uniroma3.it (C.B.)

**Keywords:** AUV, drone, Takagi-Sugeno, fuzzy, inference

## Abstract

The paper shows the steps for the preliminary studies of an AUV for shallow water: the first part illustrates the vehicle architecture and the philosophy that permeates the various design choices. In the second part illustrates an innovative method for increasing longitudinal stability based on Takagi-Sugeno (T-S) Fuzzy Inference System: it saves a lot of computational time and, by simplifying the calculation, it is also suitable for remarkably simple computers such as Arduino. in the third part is simulated the behavior of the AUV: thanks to the data taken from the previous hydrodynamic simulation, we can establish the behavior of its longitudinal stability and the computational savings due to the T-S method.

## 1. Introduction

The purpose of the paper is to study a drone capable of autonomous exploration of the sea. The vehicle differs from the others because from the environmental point of view it is designed for shallow waters: areas that are exceedingly difficult to reach with ordinary autonomous underwater vehicles (AUVs). It is imagined and so designed to perform many activities currently neglected because expensive to execute with traditional systems as, e.g., marine traffic control missions, monitoring of sandy/rocky coast, search and tracking of schools of fish, control of oil pipelines and submarine cables. For these missions it is necessary to have small vehicles but with extremely powerful engines, of compact architecture: most of the existing drones only partially satisfy any of these requirements.

The study went through several phases: outline and detailed drawing, design of the control and attitude system. Since the classic algorithms are very time consuming, one of the main innovative objectives of this part of the work is to find “light” or simplified algorithms that allow the use of simple, easily reprogrammable and inexpensive systems such as Arduino thought the application of fuzzy logic to the attitude control system [1].

Our work is divided into three main parts: in the first part we describe the general architecture design of an underwater drone for shallow water. The second part illustrates a mathematical method to increase the longitudinal stability control system which saves computational effort and is based on Takagi-Sugeno Fuzzy Inference System to use small computing systems to make development flexible and simple. In the last part, the fluid dynamic simulation of the CAD model it gives us important data that we then use for the simplified calculation procedures and algorithms that we will later simulate in real conditions.

### State of the Art

Sampling the oceans has traditionally been conducted from ships: the first global oceanographic research cruise was carried out with the ship HMS “Challenger” which led to a great number of discoveries like the mid-Atlantic ridge or the Challenger Deep in the Mariana Trench to name only a few. It took over 23 years to compile the results from this cruise: obviously, these are times that are no longer acceptable today [1,2,3,4].

**Figure 1:**
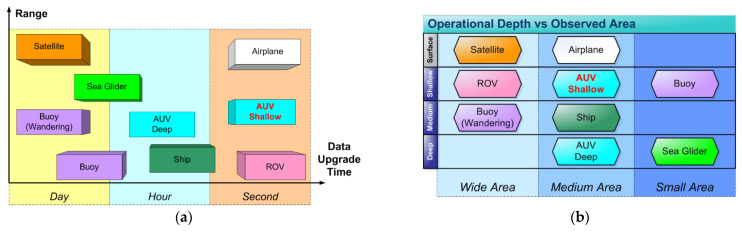
In these two figures are presented the most important classes of vehicles for sea exploration and their performances (**a**) range vs. data upgrade time; (**b**) operational depth vs. observed area.

Autonomous vehicles today represent the spearhead of underwater exploration systems. After the pioneering era of diving bells and bathyscaphe (which endangered the life of the human crew), submarine research immediately turned to automatic systems: useful robots that can carry out even the most difficult and risky missions. It is also necessary to note that we have also relied on non-proximity exploration systems such as airplanes and, above all, satellites with a payload optimized for observations on seas and oceans. The ROVs (Remote Operate Vehicle) were the first semi-autonomous vehicles used for the exploration of the deep waters but the presence of the umbilical cord places great limits both to maneuverability and to the maximum reachable depth; on the other hand, they have the advantage of sending sensor data in real time: often, however, the cost of the support vessel makes them highly uneconomic. The buoys (lagrangian and semi-lagrangian) present themselves as excellent means of exploration: unfortunately, their ability to move is influenced only by external factors such as currents and tides. In the last ten years, underwater gliders have made great strides: they allow the exploration of large volumes of water for extremely long times: unfortunately, their intrinsic dynamics do not allow them to be used near coastal shallows (see Figure 1a for comparison) [5,6,7,8].

The AUVs have established themselves for their flexibility of use, given that the technology of the driving and control systems has become very advanced. These vehicles can explore large areas in complete decision-making autonomy, complete difficult missions by counting an advanced artificial intelligence that allows them to no longer be banal executors of orders but sometimes, they are able to make creative and sophisticated decisions. This obviously within the limits of their programming and setting.

Historically, the development of these devices has been severely held back by inadequate technology and by the difficulties, including economic ones, linked to field tests of AUVs. Today, everything related to drones is in turmoil, design, controls, sensors because modern electronic technologies make it possible to obtain previously unimaginable results but, the research and the development has been principally focused on AUVs for large offshore cruises, developing several interesting prototypes [9,10]. On the contrary, both because the technological challenges for deep water AUVs is more stimulating and because certain activities are more easily carried out by divers or wire-guided ROVs, the research activity for specialized drones in shallow waters was not very intense, so neglecting all the exigencies of this world in which there is the most part of marine life and marine biodiversity which are strongly affected by the impact of human activities and of the climate change [11,12]. Even other human activities could be strongly supported by these devices, some more economically important, as the monitoring of pipelines so as submarine power lines, some emerging, as the monitoring of the coast to contrast the drug traffic or the human trafficking, some humanly important such as underwater archeology. Currently, all these activities are or occasionally performed or scheduled but never are continuously performed while it would be allowed by an autonomous drone. The use of autonomous underwater vehicles (AUV’s) provides flexibility and scalability in conducting seabed surveys overcoming many of the objective limitations. Some AUVs for shallow water have been already realized as, e.g., the Sirius AUV realized by the University of Sydney [13], the Woods Hole Oceanographic Institute realized the system called ABE [14] and the Girona 500 AUV developed at the University of Girona [15]. These systems have been happily tested in depths greater than 10 m, but show struggle to perform in shallower environments moreover their dimensions oblige to use a significant logistic support as vessels at the surface for deployment and recovery.

An already existing platform, the Starbug Mk3, has been specifically adapted to meet the exigencies of shallow water giving live to the model Starbug X. It is a small and portable AUV that can be deployed even from small inflatable vessels [16,17], but its intrinsic performance prevents a use for long measurement autonomous campaign.

Cause their use, the longitudinal stability of the AUVs is really important and a study on it typically accompanies the design of a new AUV [18,19]. 

Unfortunately, all these analyses are tailored for the specific AUV under study because they are strongly influenced by the shape and by the weight distribution of the specific device.

Our vehicle has the task to explore coastal areas full of obstacles (see Figure 1b for comparison) [20,21,22,23] and for longer lasting measurement cruises and its design is accompanied by a specific longitudinal-stability analysis.

## 2. Materials and Methods

The need to face shallow seabed and turbulent waters led us to the idea of developing a peculiar and completely new project. The architecture of our project is therefore far from all the previous ones due to the use of technical solutions which, although not original in the sense that we cannot claim authorship (e.g., the annular wing), have never been concentrated in one vehicle. Very tapered thin wings, widely used for similar drones, have proved completely unsuitable for our environment. The same can be said for the power installed on board: we need two counter-rotating propellers to overcome local speed peaks due to the turbulent state of the sea near straits or areas full of rocks. The project solutions are detailed below, explaining the choice of each part of the architecture [24,25,26,27,28].

### 2.1. The Vehicle

**Figure 2:**
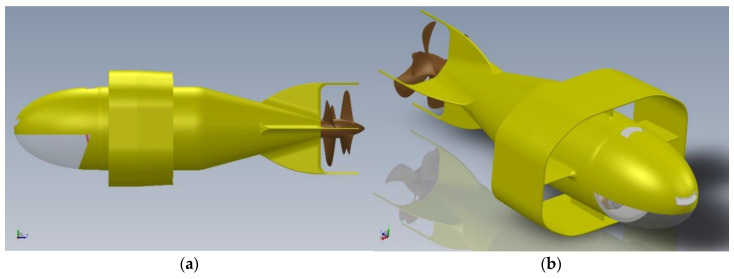
Two views of the drone (**a**) port side view; (**b**) starboard prospective view.

The underwater vehicle was named Albacore (Thunnus Alalunga) due to the extreme similarity in both size and shape with the tuna well widespread in the Mediterranean: it was designed for use in shallow, high turbulence waters, in the presence of natural obstacles (rocks and shoals) but also wrecks or breakwaters, etc. (See Figure 2a,b). For all these reasons we decided to equip it with two powerful engines that operate counter-rotating propellers and an elliptical wing, sturdy and stiff.

The estimated general characteristics and performances of the vehicle are shown in the following Table 1.

The tasks of the vehicle are several and various: first of all the surveillance of fish schools and fishing operations (thanks to the neural network) then it can monitor a well-defined area, being able to detect oil spills or biological contamination.

The vehicle is also able to partially emerge as an “autonomous periscope” for the purpose of optically monitoring short-range ship traffic.

**Figure 3:**
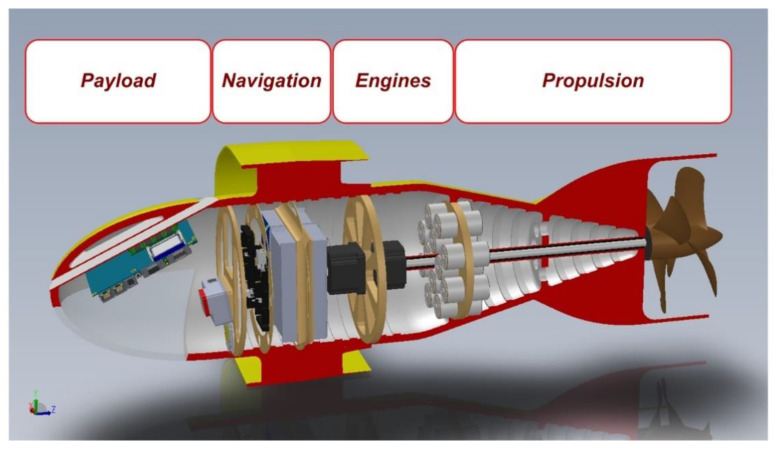
Section of AUV Albacore.

The architecture of the AUV is extremely essential: a fuselage supports an annular wing and is propelled by two counter-rotating propellers (see Figure 3).

#### 2.1.1. The Fuselage

The fuselage of the Albacore is roughly cylindrical, composed of milled aluminum 6061 class: in the front, we have designed an elliptical radome act to contain the payload that consists on several biochemical sensors arranged in a “nostril” that has the purpose of protecting the instrumentation without exposing it directly to the outside.

In the lower section, there is a transparent porthole in polymethylmethacrylate (Plexiglas): it is the window for the camera (GoPro class) and the relative lighting system.

The central part supports the supports of the elliptical wing and is further stiffened by a series of internal battens. The terminal cone (this too stiffened in the same way) supports the fletching and the thrust of two counter-rotating propellers.

The fuselage is composed by four coaxial cylindrical compartments (or bays):

- Payload bay
- Navigation bay
- Engines bay and
- Propulsion bay.

#### 2.1.2. The Payload Bay

**Figure 4:**
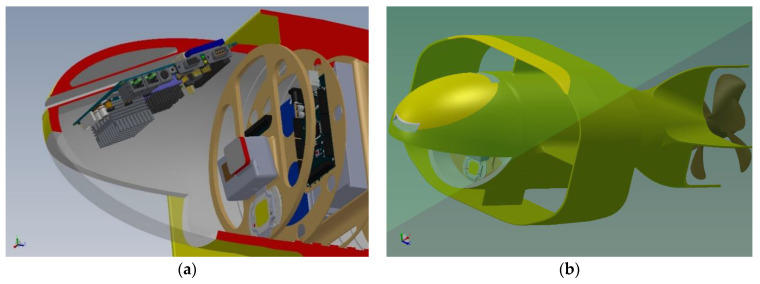
(**a**) The payload and navigation bay: cutaway side view; (**b**) drone in “nose up” attitude: prospective view.

**Figure 5:**
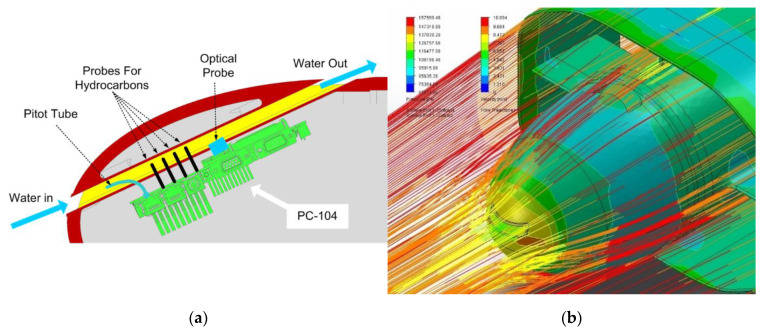
(**a**) Nostril: port cutaway side view; (**b**) simulation of the flow of water in the interference area between the nostril outlet and the annular wing.

The Payload Bay is, in essence, a “radome”, which contains the “nostril” (see Figure 4) whose channel in turn houses the chemical and biological sensors: the data collected are managed by a PC-104 computer card, which also has the task of sending them to the central computer (Arduino). It was decided to incline the “nostril” by 20°: after a series of hydrodynamic simulations, we decided to place it at 20° as the discharge flow would not have involved the annular wing, worsening its performance (see Figure 5b).

Underneath there is a large window that allows a digital camera (Go-Pro class) an excellent FOV (Field of View): this also allows the recognition of underwater objects or ships on the surface. The lighting of the scene is provided by a flat LED of 10^6^ candles, as it is intended for use in murky port waters. The LED is mounted on a bulkhead and is placed on the shoulder of the camera which dissipates the heat generated.

#### 2.1.3. The Nostril in Detail

The “nostril” is nothing more than a channel in which the water that laps the drone is forced to pass: in this flow, several sensors are immersed that analyze the water so that they can operate correctly while being sheltered. In fact, they were not placed on the external surface of the hull because, being operated by unskilled personnel, they could suffer shocks, breakages, or improper handling (see Figure 5a). The first sensor present is the pitot tube, also known as pitot probe: is a flow measurement device used to measure fluid flow velocity.

Later we have provided a series of sensors to detect the different types of hydrocarbons, in order to detect the “oil spills”. The payload is a “proposal”: we imagined the use of the drone as a sea contamination detector. The probes can be easily replaced with other “ad hoc” ones.

Finally, we have provided a laser opacimeter to measure the degree of transparency of sea water.

The sensors produce a large mass of data which then must be correlated not with time but with the position and attitude of the drone: their processing is delegated to a PC-104 card which will then also take care of data storage. In addition, many sensors are equipped by the parent company with dedicated software to manage the output of you, which runs only on a PC. It is singular that a higher computing power is required than that necessary for navigation which, instead, is delegated to a simple Arduino (redundant).

#### 2.1.4. The Navigation Bay

The Navigation Bay contains two Arduino units: due to their quality level COTS (Commercial Off-The-Shelf), it was decided to put them in Main and Redundant configuration. The second unit (redundant) is placed in “hot stand by” despite being fed and while managing the same data flow, it is not called to play the role of OBDH (On Board Computer and Data Handling) as instead the Main Unit does: this allows, in the event of a malfunction, to take over the latter in a completely transparent manner to the rest of the devices to which they are interfaced (see Figure 4a). The bay also contains the two main rechargeable batteries: one supplies power to the ODBH and the other to the payload. The differentiation was necessary because, in the event of a serious failure of the first battery, the second, disconnecting all non-essential services, can supply the energy needed by the Arduino computer to be able to lead the vehicle to the surface and to manage any recovery procedures.

#### 2.1.5. The Engine Bay

**Figure 6:**
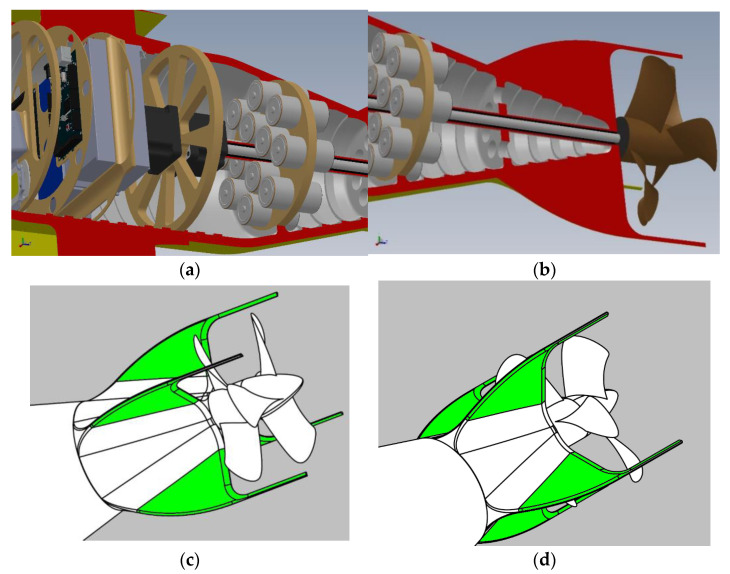
Drone cutaway side view (**a**) engines bay; (**b**) propulsion bay. Fins arrangement: the movable parts are green. Prospective view: (**c**) port side (**d**) starboard side.

The engine bay contains two identical but counter-rotating electric motors (CW and CCW) which in turn operate the two propellers, also these counter-rotating (Figure 6a). The movement is transmitted by two concentric drive shafts: the first (CW) is internal and moves the propeller at the end, the second (CCW) is hollow and allows the rotation of the first and moves the propeller closer to the hull. Due to the length of the drive shafts, two bearings were placed to attenuate any vibrations, one at the auxiliary battery cluster and another near the tail.

#### 2.1.6. The Propulsion Bay

The propulsion bay contains first and foremost the battery cluster, the drive shafts of the engines, the fletching and the two counter-rotating propellers. The battery cluster is composed of a canister that supports 12 “D” type accumulators of a completely different technology compared to the two main batteries so that, given the same environment, it has a completely different reliability (electromechanical degradation) response. Thanks to a small engine, it is possible to slide the chassis backwards so that the center of mass of the vehicle moves quite far from the hydrostatic center and so the hull can assume the “nose up” position for biochemical measurements (see Figure 6b).

The cruciform fins (see Figure 6c,d) have no dihedral and have been prolonged to act as a guard for the propellers, thus preventing them from being sized in the presence of tufts of algae or wandering nets. They are fully mobile (full floating) whose movement regulates the direction of the drone. The fulcrum of the mobile surface is placed beyond the pressure center: to restore the stability, the appendages which serve to protect the propellers also have the function of “dynamic balancing” of the control surface. Finally, the propellers are counter-rotating to counteract the strong torque of the engines, which are especially slow-moving because we are in the absence of a large wingspan that can counteract them. The terminal propeller has an angle of attack (AoA) greater than the previous one to have the same performance as the previous one, being lapped by a flow already in rotation.

#### 2.1.7. The Wing

Following a careful study, an elliptical annular wing was chosen for the vehicle: the peculiarity of the configuration was dictated by extremely strict requirements. First of all, with this solution we have practically halved the wingspan, greatly reducing the moment of inertia on the longitudinal axis: this apparent “lack of stability” is largely compensated for the presence of spoilers that guarantee the vehicle’s dynamic stability.

One of the possible applications of the AUV is that of the underwater inspection of fishing nets, submerged systems and submarine cables: the fact of having a ring-shaped wing guarantees the fact that it does not get caught in possible underwater obstacles.

Among the main requirements, it was considered that the vehicle can be used by unskilled personnel with equipment not specially adapted: it will be sufficient, therefore, to be able to set sail on board, to have a simple winch: in this case the wing has been strengthened to operate as a “bumper” and withstand without damage possible minor bumps against the ship’s rail. Finally, the elliptical annular wing gives the vehicle great dynamic stability, a modest induced resistance, a dimensional compactness: this is supported by four cross-shaped bracing that also act as a further element of stability.

### 2.2. Dynamic Force Balance

In this section, we consider the dynamic balance of forces on the vertical plane (X, Y): in these conditions the drone proceeds at a constant speed, in the discussion the variation of density and viscosity of the water with the variation of the depth will not be considered, nor of the relative variation in propeller efficiency. We will consider these constant elements with reasonable approximation in a non-negligible depth interval [29,30].

**Figure 7:**
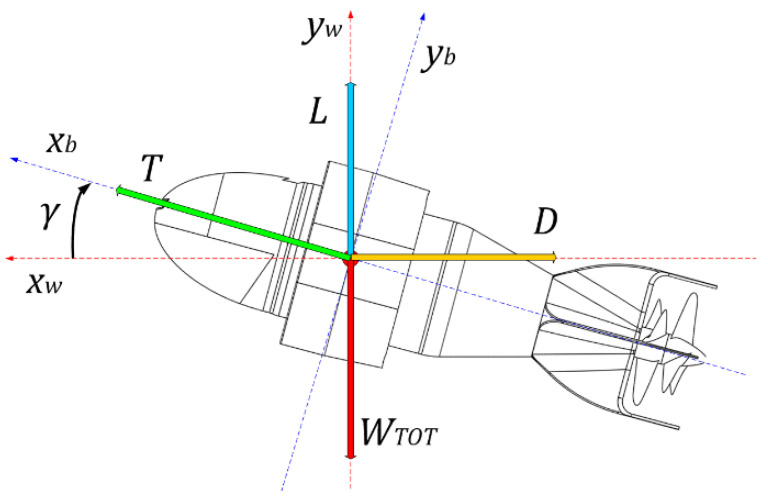
AUV Albacore: force balance on the XY plane.

The drone emerging at constant speed (see Figure 7): *x_b_* and *y_b_* axis are referred to the body of the drone*, x_w_* and *y_w_* are *absolute* axis.

At the equilibrium, the balance of the forces referred to *x_w_* and *y_w_* axis are, at constant speed:

(1) $$\{\begin{array}{l} 0=T\cos\gamma -D\\ 0=T\sin\gamma +L-W_{tot} \end{array}$$

where

T: thrust (due to the propellers)
D: drag (due to the shape of the vehicle)
v: drone relative speed (referred to water)
L: lift (due to the wing)
γ: angle of attack
$W_{tot}$: total weight

The complete expression for the *drag* is:

(2) $$D=\frac{1}{2}\rho v^{2}SC_{D}$$

where:

ρ: seawater density (average 1.025 kg/L)
S: drone wing surface
v: drone relative speed (refer to water)
$C_{D}:$ coefficient of drag

According to Taylor’s method, the last member can be separated in:

(3) $$C_{D}=C_{D_{0}}+C_{D_{\gamma }}\gamma$$

where:

$C_{D_{0}}:$ coefficient of drag at γ = 0
$C_{D_{\gamma }}:$ coefficient of drag at γ ≠ 0

so the Equation (2) becomes:

(4) $$D=\frac{1}{2}\rho v^{2}S\left(C_{D_{0}}+C_{D_{\gamma }}\gamma \right)$$

The expression for the lift is:

(5) $$L=\frac{1}{2}\rho v^{2}SC_{L}$$

where:

$C_{L}$: coefficient of lift

According to Taylor’s method as per Equation (3):

(6) $$C_{L}=C_{L_{0}}+C_{L_{\gamma }}\gamma$$

where:

$C_{L_{0}}:$ coefficient of lift at γ = 0
$C_{L_{\gamma }}:$ coefficient of lift at γ ≠ 0

so the Equation (5) becomes:

(7) $$L=\frac{1}{2}\rho v^{2}S\left(C_{L_{0}}+C_{L_{\gamma }}\gamma \right)$$

For the weight we have

(8) $$W_{tot}=W_{DW}-B_{GB}$$

where

$W_{DW}:\;$*dry* weight of the drone
$B_{GB}:$ buoyancy of the drone

so, for the Equation (1) we have:

(9) $$\{\begin{array}{l} 0=T\cos\gamma -\frac{1}{2}\rho v^{2}S\left(C_{D_{0}}+C_{D_{\gamma }}\gamma \right)\\ 0=T\sin\gamma +\frac{1}{2}\rho v^{2}S\left(C_{L_{0}}+C_{L_{\gamma }}\gamma \right)-W_{DW}+B_{GB} \end{array}$$

Now we evidence the thrust:

(10) $$\{\begin{array}{l} T\cos\gamma =+\frac{1}{2}\rho v^{2}S\left(C_{D_{0}}+C_{D_{\gamma }}\gamma \right)\\ T\sin\gamma =-\frac{1}{2}\rho v^{2}S\left(C_{L_{0}}+C_{L_{\gamma }}\gamma \right)+W_{DW}-B_{GB} \end{array}$$

So we have:

(11) $$\{\begin{array}{l} T=\frac{+\frac{1}{2}\rho v^{2}S\left(C_{D_{0}}+C_{D_{\gamma }}\gamma \right)\;}{\cos\gamma }\\ T=\frac{-\frac{1}{2}\rho v^{2}S\left(C_{L_{0}}+C_{L_{\gamma }}\gamma \right)+W_{DW}-B_{GB}}{\sin\gamma } \end{array}$$

Upper and lower member are the same, so:

(12) $$\frac{+\frac{1}{2}\rho v^{2}S\left(C_{D_{0}}+C_{D_{\gamma }}\gamma \right)\;}{\cos\gamma }=\;\frac{-\frac{1}{2}\rho v^{2}S\left(C_{L_{0}}+C_{L_{\gamma }}\gamma \right)+W_{DW}-B_{GB}}{\sin\gamma }$$

Now, in order to isolate the angle of attack:

(13) $$\frac{\sin\gamma \;}{\cos\gamma }=\;\frac{-\frac{1}{2}\rho v^{2}S\left(C_{L_{0}}+C_{L_{\gamma }}\gamma \right)+W_{DW}-B_{GB}}{+\frac{1}{2}\rho v^{2}S\left(C_{D_{0}}+C_{D_{\gamma }}\gamma \right)}$$

Then

(14) $$\tan\gamma =\;\frac{-\frac{1}{2}\rho v^{2}S\left(C_{L_{0}}+C_{L_{\gamma }}\gamma \right)+W_{DW}-B_{GB}}{+\frac{1}{2}\rho v^{2}S\left(C_{D_{0}}+C_{D_{\gamma }}\gamma \right)}$$

In case of “straight and level” trajectory we have γ = 0 so the expression becomes

(15) $$0=\;\frac{-\frac{1}{2}\rho v^{2}SC_{L_{0}}+W_{DW}-B_{GB}}{+\frac{1}{2}\rho v^{2}SC_{D_{0}}}$$

and

(16) $$0=\;-\frac{1}{2}\rho v^{2}SC_{L_{0}}+W_{DW}-B_{GB}$$

Posing

(17) $$\kappa =\;\frac{1}{2}\rho SC_{L_{0}}$$

we have:

(18) $$\kappa \cdot v^{2}=W_{DW}-B_{GB}$$

so for the speed:

(19) $$v=\sqrt{\frac{W_{DW}-B_{GB}}{\kappa }}$$

**Figure 8:**
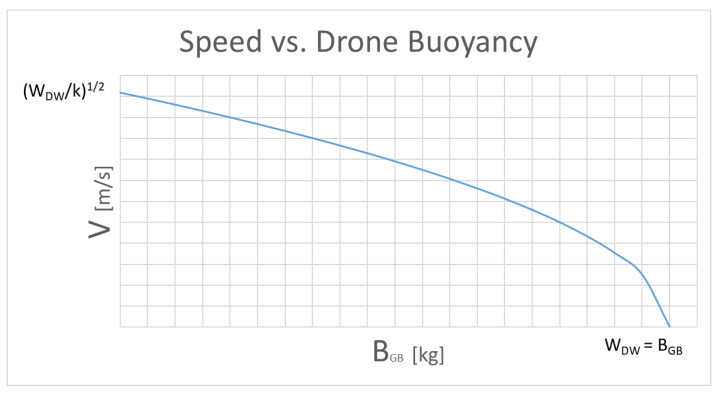
Qualitative trend of the function Speed vs. drone buoyancy.

In, the graph in Figure 8, we see the trend of the function:

The limits for v are:

(20) $$0<v<\sqrt{\frac{W_{DW}}{\kappa }}$$

the speed goes from zero to the maximum: this does not mean that the drone cannot go at higher speeds but only that it is the limit for straight and level “flight”. To reach higher speeds in horizontal paths it is necessary to choose negative angles of attack because the lift of the wing would bring the vehicle upwards.

The limits for $B_{GB}$ are:

(21) $$0\;<B_{GB}<W_{DW}$$

The variation $B_{GB}$ of buoyancy is obtained by means of a small external bladder which is filled and emptied of oil if necessary, by means of a small electric pump. Its limits are absolutely evident: a bladder that gives a hydrostatic thrust greater than the weight itself would lead the drone to float on the surface without construct.

The zero limit, on the other hand, can be overcome by appropriately ballasting the drone and obtaining a negative buoyancy: even in this case, we will have that the vehicle is over ballasted and would sink directly. This type of set-up is allowed for a sub-glider but not for a classic drone.

**Figure 9:**
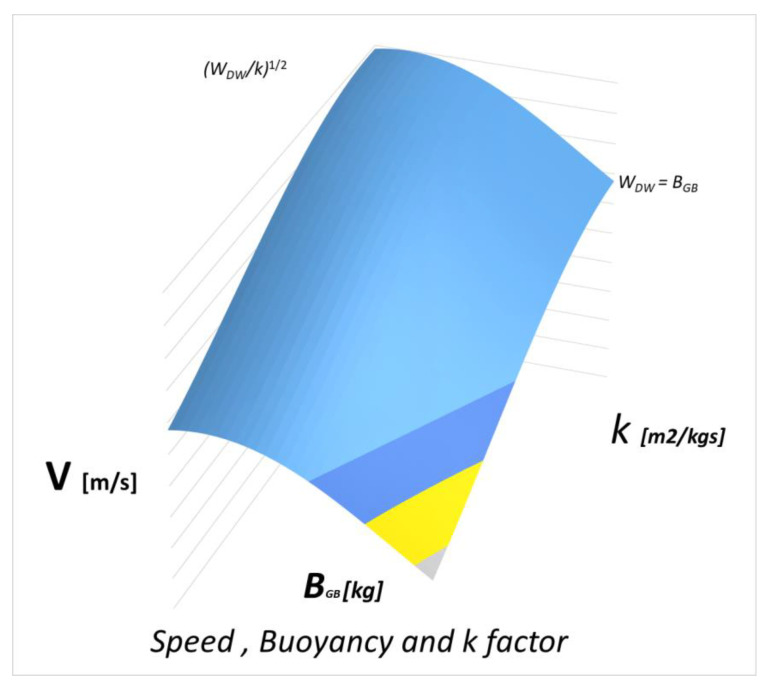
Qualitative trend of the function speed, drone buoyancy and k factor.

In Figure 9 we see the trend composed of the speed, the buoyancy, and the k-factor: we see that the curve decreases as *k* increases but not linearly as there are non-linear parameters inside [31].

### 2.3. TS Convergence

Now we apply the Takagi-Sugeno (T-S) convergence method to evaluate the longitudinal stability of our drone. Longitudinal stability is extremely important as it affects the motion and attitude of the vehicle. Its resolution in “closed” (exact) form is not always possible and it requires a high number of mathematical steps and this, in addition to absorbing a large slice of computing power, imposes a lower dynamic to the “flight envelope” of the vehicle [32].

#### 2.3.1. General Conditions

The longitudinal stability depends on few equations: the wrong combination of these critical parameters leads to the drone’s instability. We will use a simplified mathematical model of the vehicle: this is allowed by to the low dynamics of the system and to by the high density (and viscosity) of the fluid in which it is immersed.

The expression for the *lift* is (see Equation (7)):

(22) $$L=\frac{1}{2}\rho v^{2}SC_{L}=\frac{1}{2}\rho v^{2}S\left(C_{L_{0}}+C_{L_{\gamma }}\gamma \right)\;$$

where:

ρ = seawater density (average 1.025 kg/L)
S = drone surface
v = drone relative speed (refer to water)
$C_{L}$ = coefficient of lift

**Figure 10:**
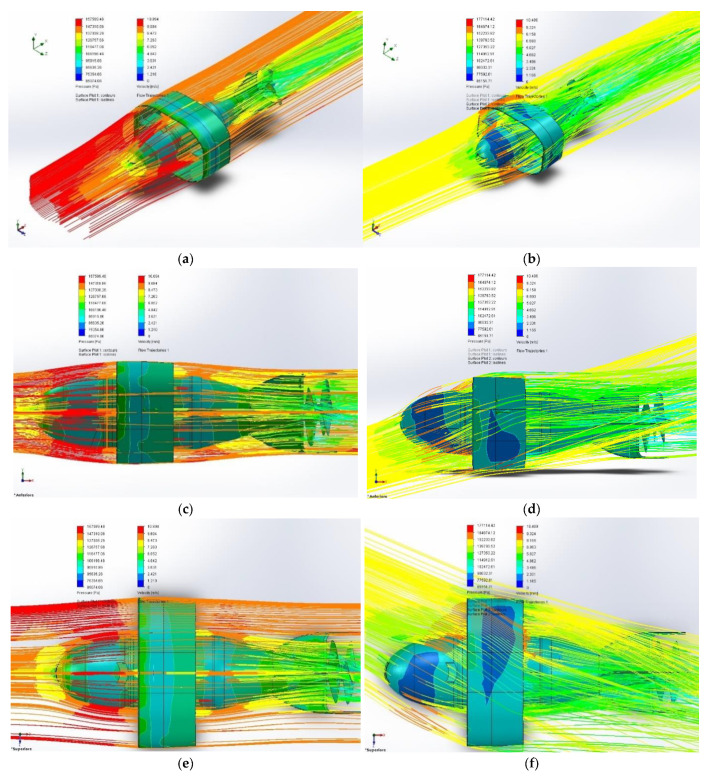
Three views of SolidWorks^®^ simulation of the drone (**a**,**c**,**e**) angle of attack null; (**b**,**d**,**f**) angle of attack not null (15° xy plane, 15° xz plane).

We evaluated the vehicle’s coefficient of lift $C_{L}$ by inserting the CAD model (developed with SolidWorks^®^) into its fluid dynamics application (see Figure 10), changing the angle of attack [33,34].

This was necessary as it is not possible to carry out a satisfactory fluid dynamic analysis by examining only the annular wing and ignoring its interference with the fuselage. Furthermore this type of simulation allowed us to examine in detail the hydrodynamic behavior with various angles of attack has been started. This is because we cannot in any case verify neither the tail effect nor the strongly tapered fuselage effect.

Our CD model was inserted into a constant velocity water flow (V_∞_ = 10 m/s) where $C_{L}$ and $C_{D}$ were calculated for a large number of angles of attack.

**Figure 11:**
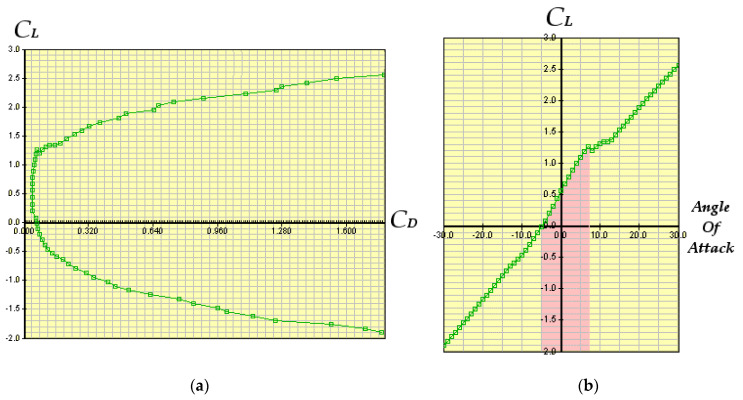
The whole drone hydrodynamic behavior (**a**) coefficient of lift vs. coefficient of drag; (**b**) coefficient of lift vs. angle of attack: the zone evidenced in red is linear.

The result of the research has provided the two graphs represented in Figure 11a,b.

#### 2.3.2. Fuzzy Stabilization

The red zone shows the linear interval for the angle of attack γ (Figure 11b).

The Equation (22) can be linearized expressed as:

(23) $$L=\frac{1}{2}\rho v^{2}SC_{L_{0}}\;+\frac{1}{2}\rho v^{2}SC_{L_{\gamma }}\gamma =\frac{1}{2}\rho v^{2}SC_{L_{0}}\;+\frac{1}{2}\rho v^{2}Sk\gamma =\frac{1}{2}\rho v^{2}S\left(C_{L_{0}}+k\gamma \right)\;$$

The speed is between the stall speed and the maximum speed.

We can express the longitudinal stability interval as:

(24) $$\{\begin{array}{c} {\dot{x}}_{H}=v\;\\ L≅\frac{1}{2}\rho v^{2}S\left(C_{L_{0}}+k\gamma \right) \end{array}$$

and they can be written as:

(25) $${\dot{x}}_{M}=\left[\begin{array}{c} x_{1}\\ x_{2} \end{array}\right]=\left[\begin{array}{c} {\dot{x}}_{H}\\ L \end{array}\right]=\;\left[\begin{array}{cc} 1 & 0\\ \rho vSk\gamma & \frac{1}{2}\rho v^{2}Sk \end{array}\right]\;\cdot \left[\begin{array}{c} v\\ \gamma \end{array}\right]$$

Here v and α are nonlinear terms in the last expressions and our fuzzy variables.

So, we can express the Equation (25) as:

(26) $$\left[\begin{array}{cc} 1 & 0\\ \rho vSk\gamma & \frac{1}{2}\rho v^{2}Sk \end{array}\right]=\left[\begin{array}{cc} 1 & 0\\ z_{1} & z_{2} \end{array}\right]$$

The nonlinear terms limits are:

(27) $$\{\begin{array}{c} v\in \left[\begin{array}{cc} v_{stall} & v_{max} \end{array}\right]\\ \gamma \in \left[\begin{array}{cc} -4.5 & 8 \end{array}\right] \end{array}$$

So, Equation (26) at the limits become:

(28) $$\begin{array}{c} \begin{array}{c} max\;z_{1}\\ v,\gamma \end{array}=8\rho v_{max}Sk\\ \begin{array}{c} \begin{array}{c} min\;z_{1}\\ v,\;\gamma \end{array}=-4.5\rho v_{stall}Sk\\ \begin{array}{c} \begin{array}{c} max\;z_{2}\\ v,\;\gamma \end{array}=\frac{1}{2}\rho v_{max}{}^{2}Sk\\ \begin{array}{c} min\;z_{2}\\ v,\gamma \end{array}=\frac{1}{2}\rho v_{stall}{}^{2}Sk \end{array} \end{array} \end{array}$$

Therefore v and α can be represented by for membership functions M_1_, M_2_, N_1_ and N_2_ as follows:

(29) $$z_{1}=M_{1}\left(z_{1}\right)\cdot \left(8\rho v_{max}Sk\right)+{\;M}_{2}\left(z_{1}\right)\cdot \left(-4.5\rho v_{stall}Sk\right)\;z_{2}=N_{1}\left(z_{2}\right)\cdot \left(\frac{1}{2}\rho v_{max}{}^{2}Sk\right)+{\;N}_{2}\left(z_{2}\right)\cdot \;\left(\frac{1}{2}\rho v_{stall}{}^{2}Sk\right)$$

where

(30) $$\{\begin{array}{l} M_{1}\left(z_{1}\right)+M_{2}\left(z_{1}\right)=1\\ N_{1}\left(z_{2}\right)\;+{\;N}_{2}\left(z_{2}\right)=1\;\; \end{array}$$

The *model rules* are:

RULE #1: IF z_1_ is “*high*” AND z_2_ is “*big*” THEN ${\dot{x}}_{M}=A_{1}\cdot x_{M}$
RULE #2: IF z_1_ is “*high*” AND z_2_ is “*small*” THEN ${\dot{x}}_{M}=A_{2}\cdot x_{M}$
RULE #3: IF z_1_ is “*low*” AND z_2_ is “*big*” THEN ${\dot{x}}_{M}=A_{3}\cdot x_{M}$
RULE #4: IF z_1_ is “*low*” AND z_2_ is “*small*” THEN ${\dot{x}}_{M}=A_{4}\cdot x_{M}$

where:

(31) $$A_{1}=\left[\begin{array}{cc} 1 & 0\\ max\;z_{1} & max\;z_{2}\\ z_{1}\in \;high & z_{2}\in big \end{array}\right]=\left[\begin{array}{cc} 1 & 0\\ 8\rho v_{max}Sk & \frac{1}{2}\rho v_{max}{}^{2}Sk \end{array}\right]$$

(32) $$A_{2}=\left[\begin{array}{cc} 1 & 0\\ max\;z_{1} & max\;z_{2}\\ z_{1}\in high & z_{2}\in small \end{array}\right]=\left[\begin{array}{cc} 1 & 0\\ 8\rho v_{max}Sk & \frac{1}{2}\rho v_{stall}{}^{2}Sk \end{array}\right]$$

(33) $$A_{3}=\left[\begin{array}{cc} 1 & 0\\ max\;z_{1} & max\;z_{2}\\ z_{1}\in low & z_{12}\in big \end{array}\right]\;=\left[\begin{array}{cc} 1 & 0\\ -4.5\rho v_{stall}Sk & \frac{1}{2}\rho v_{max}{}^{2}Sk \end{array}\right]$$

(34) $$A_{4}=\left[\begin{array}{cc} 1 & 0\\ max\;z_{1} & max\;z_{2}\\ z_{1}\in \;low & z_{12}\in small \end{array}\right]=\left[\begin{array}{cc} 1 & 0\\ -4.5\rho v_{stall}Sk & \frac{1}{2}\rho v_{stall}{}^{2}Sk \end{array}\right]$$

Now, ${\dot{x}}_{M}$ can be derived out of defuzzifcation process as:

(35) $${\dot{x}}_{M}=h_{1}\left(z\right)\;A_{1}\cdot x_{M}\;+\;h_{2}\left(z\right)\;A_{2}\cdot x_{M}\;\;+\;h_{3}\left(z\right)\;A_{3}\cdot x_{M}\;\;+\;h_{4}\left(z\right)\;A_{4}\cdot x_{M}$$

where

(36) $$\{\begin{array}{c} \begin{array}{c} h_{1}\left(z\right)\;=\;M_{1}\left(z_{1}\right)\;\times \;N_{1}\left(z_{2}\right)\\ h_{2}\left(z\right)\;=\;M_{1}\left(z_{1}\right)\;\times \;N_{2}\left(z_{2}\right) \end{array}\\ \begin{array}{c} h_{3}\left(z\right)\;=\;M_{2}\left(z_{1}\right)\;\times \;N_{1}\left(z_{2}\right)\\ h_{4}\left(z\right)\;=\;M_{2}\left(z_{1}\right)\;\times \;N_{2}\left(z_{2}\right) \end{array} \end{array}$$

This fuzzy model exactly represents the nonlinear system in the region

(37) $$\left[\begin{array}{cc} v_{stall} & v_{max} \end{array}\right]\times \;\left[\begin{array}{cc} -4.5 & 8 \end{array}\right]$$

in the v, γ space.

**Figure 12:**
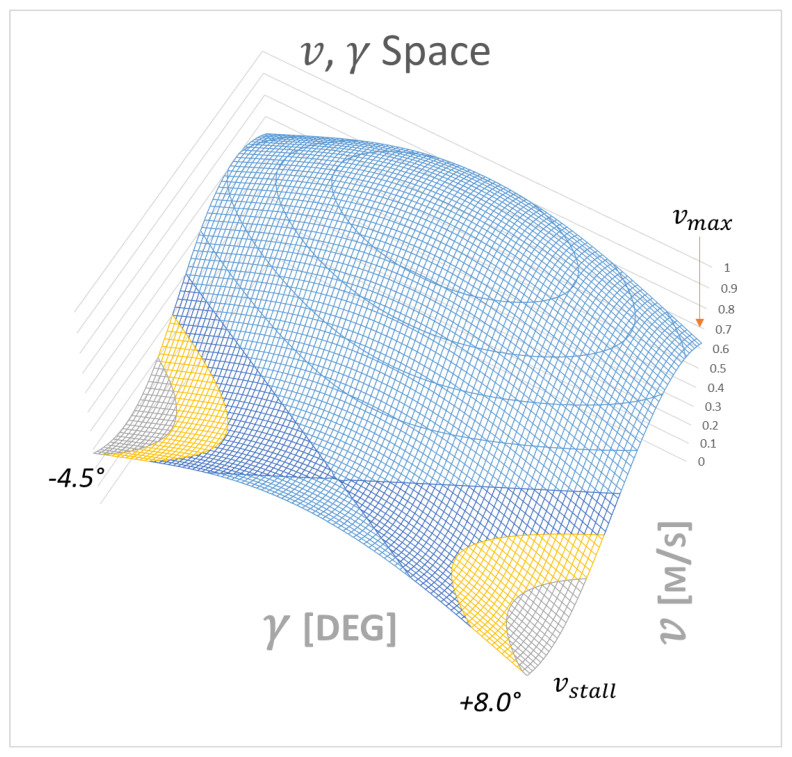
The T-S solution in v, γ space dominion.

As shown in Equations (35) and (36), the original equations are followed exactly by the proposed fuzzy model, which therefore represents the physical domain within the bounded interval (see Figure 12) of v and γ (out of the linearization).

It is interesting that Figure 12 highlights the stability solution of the T-S method. The drone, in the first linear zone is extremely unstable or the zero position “on top of the hill” (the elliptical zone) represents the unstable balance of the wing as, at low angles of attack, the beneficial and stabilizing effect is not felt of the tail which, instead, begins to show itself in the “saddle” of stability (green area).

## 3. Results

We simulated the behavior of the drone’s navigation system by entering real data from another project to see how it would respond to our method. To do this, we obviously had to enter the static and dynamic model of the drone (center of gravity, moments of inertia on the main axes, fluid dynamics analysis, etc.): for this, we had to absolutely detail our project and study its behavior once immersed in a movement, as was shown in the initial part of our work.

To estimate the error on the angle of attack, we simulated its linear and quasistatic variation in the [−4.5, 8] interval, then we evaluated the deviation (error).

**Figure 13:**
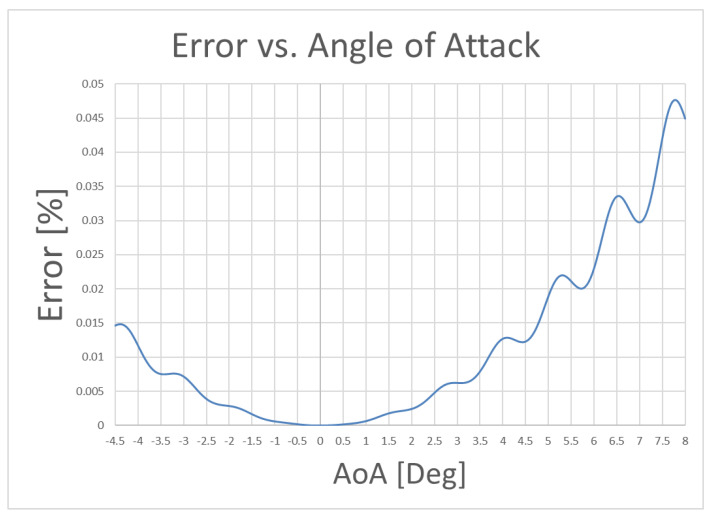
Error between the exact solution and the approximate solution vs angle of attack.

Figure 13 shows us the error that is made between the exact value that is calculated with the canonical formula and the approximation that is made with the T-S system. We see that we arrive at an extremely mild and very acceptable error in the face of a rather high calculation speed since in the second method everything is solved with a multiplication between matrices. We also see that on the error function another periodic error is “modulated” due to the sampling of the measurement which has been suitably corrected.

**Figure 14:**
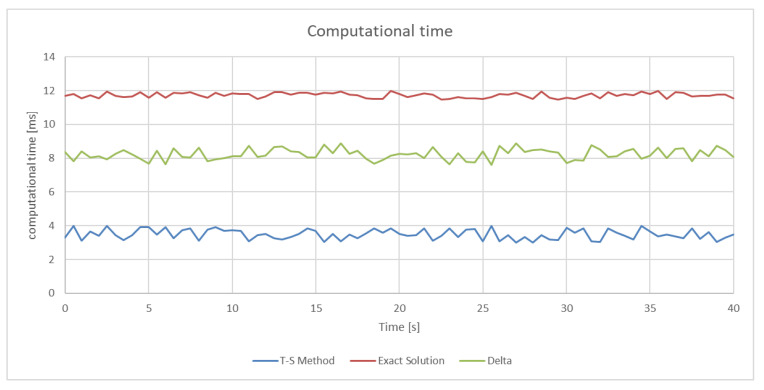
One of the results of the computational time simulation: the T-S method (blue line), the “exact” solution (red line) and the difference (green line).

Regarding the processing time, we took a “data strip” lasting 40 s and sampled every 0.5 s, evaluating and comparing the results. In Figure 14, we see the result one of the simulations: the calculation time of the “exact” solution is visible (red line), while the T-S method (blue line) allows a saving of calculation time of the order of 60%. The green line represents the time difference between the two methods.

We took 50 40-s (non-overlapping) samples of a real data strip at 50 different initial times. For each of these data samples we performed the same simulation: the real data refer to an evolving drone and therefore involve the most varied and diverse set-up situations. We then recorded the data: in particular for the delta (time difference between the two calculation methods) we made a statistical correlation with the intervals: the result (visible in the Figure) Comforts us because 83.95% of the results fall within the interval 7.91–8.70 ms.

**Figure 15:**
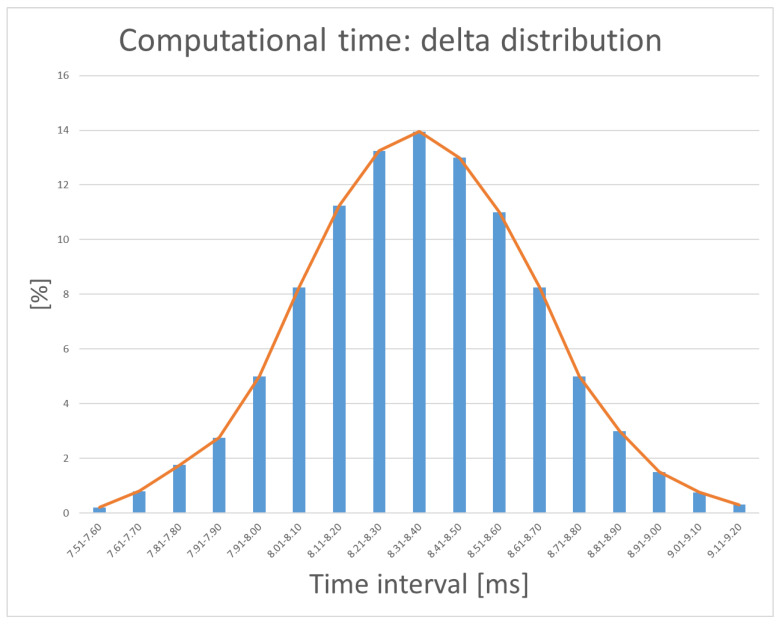
The statistical distribution of the delta computational time.

In Figure 15, we see the delta distribution through the various tests in percentage.

This saving of time and reducing the computing effort has led to use a simple Arduino system instead of other much more powerful and sophisticated computers.

## 4. Conclusions

As part of a preliminary study for a self-propelled AUV, designed for a marine environment composed of turbulent and shallow waters, we have proposed a rather compact vehicle architecture. Having chosen an annular wing, this ensures a rigid and robust structure, which is why it can also be handled by unskilled labor. The entire front part (payload bay) can be completely customized or, depending on the use, can be “loaded” with suitable sensors.

From this general model a detailed model drawn with CAD (*Solidworks*) was obtained: the model in turn was subjected to simulated fluid dynamics tests to establish its behavior in a water flow with different angles of attack (AoA).

Since one of the critical points of this vehicle is the reduced “wingspan” which leads to a certain intrinsic instability in certain attitudes, we have placed a particular study in longitudinal stability for which we have dedicated a separate section. Then we dedicated ourselves to deepen a simplified stability method so that it could be digested by a simple “Arduino-like” machine: the method for increase the longitudinal stability, which saves a lot of computational effort, is based on Takagi-Sugeno Fuzzy Inference System in order to use small computing systems and make further developments quite simple.

In the third and last part, we evaluated the possible calculation effort of a machine, inserting a series of real data and evaluating the error made in the simplification or the “cost” of truncating the calculation. Then we evaluated the machine processing times by comparing the calculation periods of both the exact method and the one based on the T-S. Both tests gave positive and extremely encouraging results for future further studies.

## Figures and Tables

**Table 1:** AUV *Albacore*: characteristics and performances (estimated).

Displacement	Length	Beam ^1^	Wingspan	Cruise Speed		Range ^2^	Endurance ^2^	Depth
59	0.920	0.22	0.445	18	34	~5700	168	200
kg	m	m	m	knots	km/h	km	h	m

^1^ Without annular wing. ^2^ At cruise speed.

## Data Availability

Not Applicable.
